# Structure‐Based Discovery of A Small Molecule Inhibitor of Histone Deacetylase 6 (HDAC6) that Significantly Reduces Alzheimer's Disease Neuropathology

**DOI:** 10.1002/advs.202304545

**Published:** 2023-11-21

**Authors:** Prasenjit Mondal, Ping Bai, Ashley Gomm, Grisilda Bakiasi, Chih‐Chung Jerry Lin, Yanli Wang, Se Hoon Choi, Rudolph E. Tanzi, Changning Wang, Can Zhang

**Affiliations:** ^1^ Genetics and Aging Research Unit, McCance Center for Brain Health, MassGeneral Institute for Neurodegenerative Disease, Department of Neurology, Massachusetts General Hospital Harvard Medical School Boston Charlestown Boston MA 02114 USA; ^2^ Athinoula A. Martinos Center for Biomedical Imaging, Department of Radiology, Massachusetts General Hospital Harvard Medical School Building 149, Charlestown Boston MA 02129 USA

**Keywords:** acetylation, Alzheimer's disease, epigenetic, HDAC6 inhibitor, neuroinflammation, phagocytosis

## Abstract

Histone deacetylase 6 (HDAC6) is one of the key histone deacetylases (HDACs) that regulates various cellular functions including clearance of misfolded protein and immunological responses. Considerable evidence suggests that HDAC6 is closely related to amyloid and tau pathology, the two primary hallmarks of Alzheimer's disease (AD). It is still unclear whether HDAC6 expression changes with amyloid deposition in AD during disease progression or HDAC6 may be regulating amyloid phagocytosis or neuroinflammation or other neuropathological changes in AD. In this work, the pathological accumulation of HDAC6 in AD brains over age as well as the relationship of its regulatory activity ‐ with amyloid pathogenesis and pathophysiological alterations is aimed to be enlightened using the newly developed HDAC6 inhibitor (HDAC6i) PB118 in microglia BV2 cell and 3D‐AD human neural culture model. Results suggest that the structure‐based rational design led to biologically compelling HDAC6i PB118 with multiple mechanisms that clear Aβ deposits by upregulating phagocytosis, improve tubulin/microtubule network by enhancing acetyl α‐tubulin levels, regulate different cytokines and chemokines responsible for inflammation, and significantly reduce phospho‐tau (p‐tau) levels associated with AD. These findings indicate that HDAC6 plays key roles in the pathophysiology of AD and potentially serves as a suitable pharmacological target through chemical biology‐based drug discovery in AD.

## Graphical abstract

Structure‐based discovery of small molecule histone deacetylase 6 (HDAC6) inhibitor PB118 shows multi‐modal mechanisms of action that lead to the attenuation of Alzheimer's disease (AD) neuropathology through clearing amyloid debris by phagocytosis, stabilizing microtubule network by elevating the expression of acetylated‐α‐tubulin, and significantly reducing tau phosphorylation and neuroinflammation. This validates the potential therapeutic application of PB118 and the significance of HDAC6 as a pharmacophore target in AD.

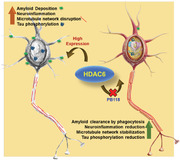

## 1 Introduction

Alzheimer's disease (AD) is a prevalent neurodegenerative disorder characterized by the formation and deposition of cerebral amyloid β (Aβ) plaques that have abundant downstream effects such as increased hyperphosphorylation of tau, neuroinflammatory events, which ultimately results in neurodegeneration and cognitive decline.^[^ ^1^, ^2^, ^3^, ^4^ ^]^ Although this amyloid cascade has been identified as a persuasive hypothesis explaining the etiology of AD and research has been ongoing for decades to develop potent therapeutics against it, the success rates are limited primarily due to its complexity. Recent reports suggest the development of novel biomarkers,^[^ ^5^ ^]^ new approaches by genetic,^[^ ^6^ ^]^ and theragnostic^[^ ^7^ ^]^ means to combat AD. We need more alternative approaches like these to find a definitive solution against this devastating disease.

Histone deacetylases (HDACs) are the epigenetic regulators that play crucial roles in eliminating acetyl groups from acetylated histone, tubulin/microtubule, and result in chromatin compaction, transcriptional repression, and destabilization of microtubule networks respectively.^[^ ^8^, ^9^, ^10^ ^]^ Among the 18 identified isotypes of HDACs, including Zn2+‐dependent HDACs (HDAC1‐11) and nicotinamide adenine dinucleotide (NAD+)‐dependent HDACs (Sirt1‐7), HDAC6 is a unique member because of its particular finger domain for binding ubiquitinated proteins, and two distinct deacetylase domains (CD1 and CD2, located at the mid and N‐terminal region, respectively). HDAC6 can deacetylate tubulin and is generally present in the cytoplasm.^[^ ^11^, ^12^ ^]^ These characteristics enable HDAC6 to control a wide range of biological functions, such as clearance of misfolded protein by degradation, immunological response, cell migration, cell proliferation, and neurological changes.^[^ ^13^, ^14^ ^]^

In recent years, there has been keen interest in establishing the relationship between HDAC6 and AD. Several studies have discovered an elevated expression of HDAC6 in the brains of AD patients,^[^ ^15^ ^]^ which results in lowering acetylated α‐tubulin levels that eventually cause dysfunctional neurons.^[^ ^12^ ^]^ Moreover, HDAC6 engages in interactions with tau protein to promote tau hyperphosphorylation, which results in neurofibrillary tangles (NFTs) formation.^[^ ^16^, ^17^ ^]^ Therapeutics potential of HDAC6 inhibitors (HDAC6is) is gaining more interest after pharmacological treatments of some selective HDAC6is in AD models show reduction of tau levels, improvement in axonal transport and restoration of learning and memory, and anti‐inflammatory activity.^[^ ^4^, ^18^, ^19^, ^20^ ^]^ However the underlying role of HDAC6 in AD needs further investigation at cellular levels for the advancement of potential drug molecules. As the pathogenesis of AD displays complex heterogeneity, including amyloid and tau pathology, as well as neuroinflammatory events, it is key to comprehensively analyze HDAC6 and HDAC6is considering these multiple molecular mechanisms. Particularly, in AD, the primary Aβ species in AD brains is the Aβ (1‐42) peptide which is generated by the sequential cleavage of transmembrane amyloid precursor protein (APP) by β‐ and γ‐secretases. Activation of pro‐inflammatory genes and consequent generation of cytokines induce when Aβ peptides bind to the cell surface receptors^[^ ^21^ ^]^ and affect neuronal damage through reactive oxygen species (ROS) dependent pathways.^[^ ^1^, ^22^ ^]^ Therefore, clearance of excess Aβ species from the extracellular brain space by phagocytosis in glial and astrocyte is crucial to regulate normal brain function and likely protective in the early stages of AD. During later stages, glial cells' impaired phagocytic function certainly plays a role in the worsening of disease outcomes, although the underlying mechanisms are yet to be understood.^[^ ^23^ ^]^ Hence, to stop plaque formation and activation of proinflammatory cytokines and related consequences, it is crucial to comprehend AD‐related molecular processes that underlie glial phagocytosis, Aβ degradation, and microtubule stabilization with probable roles of HDAC6 for the advancement of AD therapeutics. Therefore, in this work, we performed a series of chemical biology studies using disease‐related models, and specifically try to analyze the mechanistic potential in the rational design of potent and specific HDAC6is for AD. Our biological studies were designed to enlighten pathological accumulation of HDAC6 in AD animal brains and how it changes over age, as well as the association between HDAC6 regulation with AD‐associated amyloid pathogenesis and pathophysiological alterations (phagocytosis, chemokines/cytokines generation or microtubule degeneration) in detail using our newly developed HDAC6i PB118^[^ ^15^ ^]^ in the microglia BV2 cell and the 3D human neural stem cell model of AD. Collectively, the results of our current work will be well‐poised to uncover the chemical biology of HDAC6 in AD by structural‐based design and mechanistic elucidation using suitable disease models, which may provide novel insights in understanding AD and advance the drug discovery of AD.

## 2 Results and Discussion

We hypothesized that HDAC6 can be a desirable drug target for the development of potent therapeutics against AD. In this study, we have tried to enlighten the rational design, as well as to characterize molecular mechanistic aspects of HDAC6 in neuropathological changes in the brain over time, and to advance the therapeutic development of targeting HDAC6 in AD pathogenesis.

### 2.1 Pharmacophore Analysis, Design Concept, and Chemical Properties of Novel HDAC6 Inhibitor PB118

**Figure 1:**
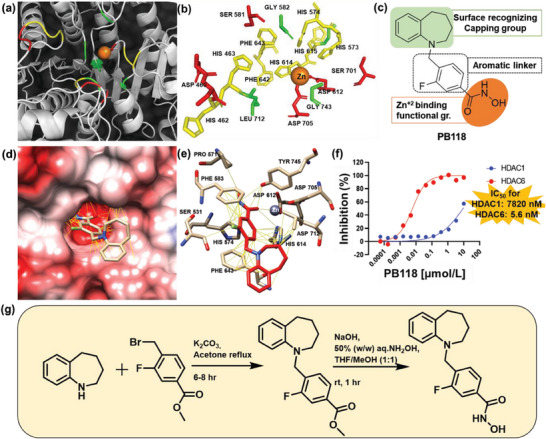
Chemical properties, and synthesis of PB118: a,b) Molecular docking and pharmacophore analysis of HDAC6 enzyme reveals several aromatic amino acids (yellow) with polar (red) and non‐polar (green) amino acids around the inhibitor binding site. c) Chemical structure of PB118, designed and developed based on pharmacophore and SAR analysis. d) Molecular docking shows good binding of PB118 with HDAC6 enzyme, interacting partners shown in e) are mostly interacting by van der Waals force and pi–pi interaction. f) HDAC inhibitory assay of PB118 with HDAC1 and HDAC6 enzyme shows >1000 times higher selectivity of PB118 towards HDAC6 makes it a potent HDAC6i. g) Synthesis route of PB118 with required reagents and reaction conditions.

The HDAC6 protein crystal structure reveals several aromatic amino acids (His 462, His 463, His 573, His 574, His 614, His 615, Phe 642, Phe 643) with polar (Asp 460, Asp 612, Ser 581, Ser 701, Asp 705) and non‐polar amino acids (Gly 582, Leu 712, Gly 743) around the inhibitory binding site (**Figure** **1**a,b). The pharmacophore analysis of selective HDAC6is from our group and others primarily reveals three domains: zinc‐binding group, surface‐recognizing capping group, and a linker between them (Figure 1c).^[^ ^24^ ^]^ We found that the capping group plays a critical role in recognizing the surface for binding and brain permeability.^[^ ^15^ ^]^ Moreover, inhibitors with large and rigid capping groups have a more favorable interactions with the hydrophobic cavity of HDAC6. We introduced a lipophilic capping group to increase the paracellular diffusion and blood‐brain barrier (BBB) permeability.^[^ ^20^ ^]^ Along with this, several capping groups were introduced and we checked the activity of all these inhibitors and found that the binding affinity of PB118 is very high towards HDAC6 (Figure 1c), which was recently reported in our previous works.^[^ ^15^ ^]^ It also revealed that PB118 exhibited good off‐target binding profile for 46 CNS‐related targets, indicating the great potential of PB118 as a therapeutic agent for AD and other brain disorders. Thereafter, we performed the molecular docking experiment to explore the interaction between PB118 and HDAC6. We have used the online docking server HDOCK^[^ ^25^ ^]^ for the docking of PB118 with an available HDAC6 protein crystal structure (PDB ID: 6THV).^[^ ^26^ ^]^ Figure 1d shows good binding of PB118 in the hydrophobic cavity of HDAC6. Interacting partners around this cavity with PB118 are shown in Figure 1e. The hydroxamate group binds to the Zn^+2^ unit and π‐π stacking interactions were observed between the aromatic linker unit of PB118 with the Phe 583, His 574, Phe 643, and His 614 aromatic amino acid residues of HDAC6 binding cavity. Furthermore, the fluorine atom on the linker was introduced to enhance the binding affinity and selectivity towards HDAC6 by forming favorable van der Waals interaction. After that, enzyme inhibition assay of PB118 was executed with different HDACs enzyme to analyze the inhibition efficiency of PB118. Results suggest that PB118 has >1000 times better selectively towards HDAC6 enzyme and has very low IC50 (5.6 nm) compared to HDAC1 (7820 nm) (Figure 1f). For the synthesis of PB118, we used 2,3,4,5‐tetrahydro‐1H‐benzo[b]azepine and methyl 4‐(bromomethyl)−3‐fluorobenzoate as the starting materials (Figure 1g). The intermediate ester was purified by silica gel chromatography and the final crude product was purified by a reverse phase C‐18 column using Combi flash and lyophilized to get a white powder. Detailed chemical characteristics including NMR, mass, HPLC purification, and yield for lead compound as well as intermediates were recently reported.^[^ ^15^ ^]^ The schematic representation of the synthesis process and NMR results for PB118 are shown in Figure S1 (Supporting Information).

### 2.2 Stability of PB118 in Physiological Condition and ADME/PK Studies

To evaluate the suitability of PB118 recapitulating an in vivo system, we performed the stability experiment with PB118 in a physiological condition. After 24 h of mixing PB118 in the human serum, there was ≈57% of compound remaining (Figure S2a, Supporting Information). We calculated the half‐life (t_1/2_) using the formula in Figure S2b (Supporting Information), which was 30.2 h. The decay constant was 0.0000063 s and the mean lifetime was 43.6 h that indicated the robust nature and suitability of PB118 for a potential therapeutic application.^[^ ^27^ ^]^ Further, to analyze the bio‐distribution of PB118, ADME studies and in vivo PK profiling of PB118 were performed that revealed 99.4% binding of PB118 with human plasma and 67.9 min of half‐life in human microsomal liver (Figure S3, Supporting Information, left). Brain/plasma study of PB118 on C57BL/6 mice (i.p 1 mg kg^−1^) followed by collection of blood and brain samples at 30 min and 1 and 4 h time points revealed BBB crossing ability of PB118 (Figure S3, Supporting Information, right).

### 2.3 Age‐Dependent Changes of HDAC6 Expression and Amyloid Deposition in AD Transgenic Animals

**Figure 2:**
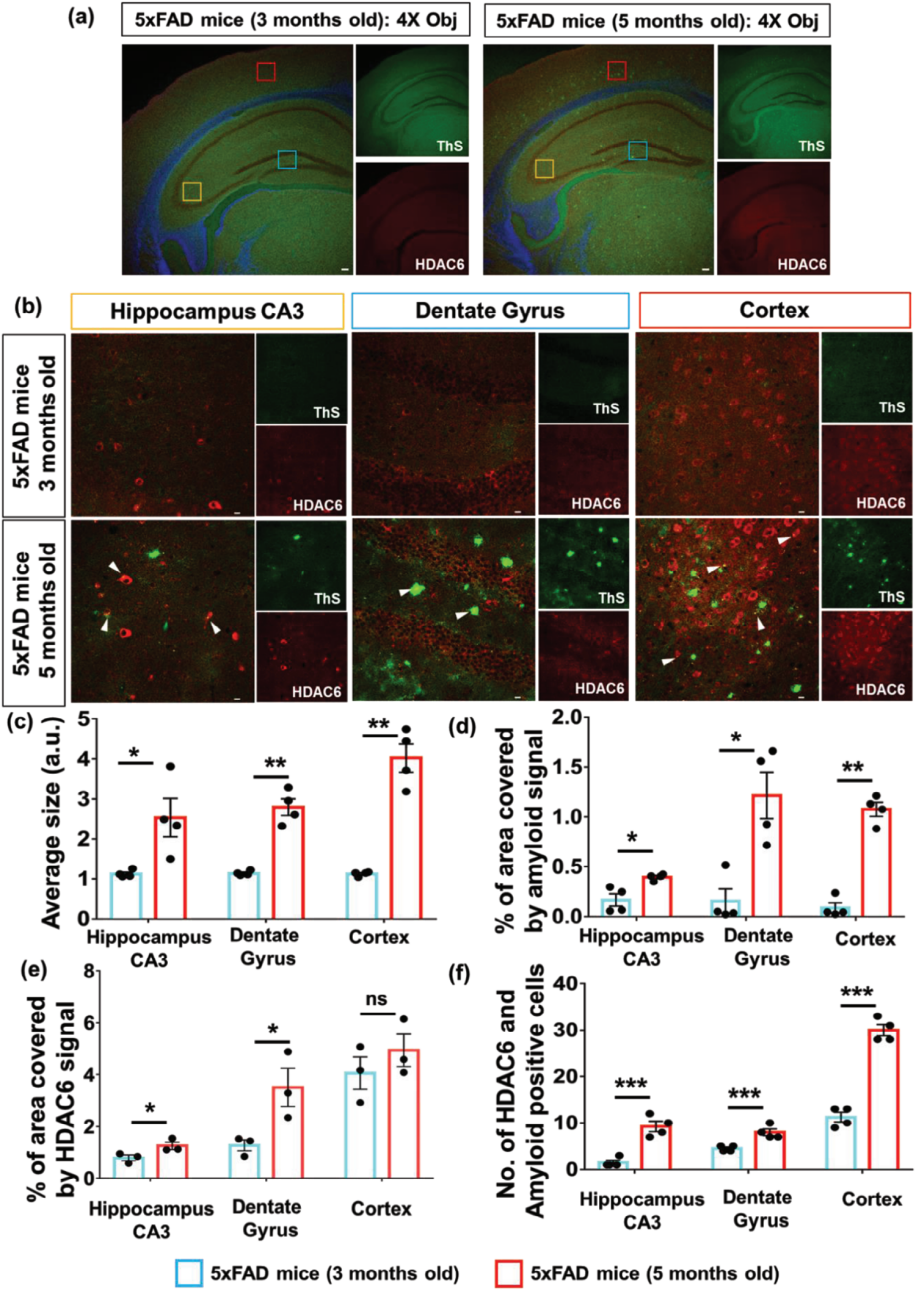
Immunohistochemistry (IHC) study to measure HDAC6 and amyloid expression in differently aged transgenic 5xFAD mice: a) Confocal microscopy images showed animal brain sections probed with HDAC6 antibody for HDAC6 and ThS for amyloid β‐sheet structure, which were captured in different channels [488, green (amyloid); 594, red (HDAC6)] using 4X objective (scale bars corresponded to 100 µm). b) Different sub‐regions of brain sections in A (colored boxes) were further magnified (40X objective) for better understanding the expressions of HDAC6 and amyloid deposition, with white arrows showing the overlapping regions (scale bar corresponded to 10 µm). Quantification and statistical analysis of images were performed in GraphPad Prism software. Image J analysis software was used to calculate average sizes (c) with area (d) of amyloid deposits and area covered by HDAC6 protein expression (e) and no. of amyloid and HDAC6 positive cells (f) in different sub‐areas of brain sections of 3 and 5‐month‐old 5xFAD mice. All the results shown were represented by mean ± SEM after performing each experiment three times. Statistical analysis was performed using a two‐tailed Student's *t*‐test: where ^*^ *p* <0.05, ^**^ *p* <0.001, ^***^ *p* <0.0001, ns = non‐significant.

We recently reported elevated expression of HDAC6 in different brain sub‐regions of transgenic mice (5xFAD) comparing wild type (WT) in our previous work.^[^ ^15^ ^]^ Here, we performed an immunohistochemistry (IHC) study with differently aged (3 and 5 months) 5xFAD mice brain sections. These sections were probed with thioflavin‐S (ThS), a dye that usually binds with the beta‐sheet (β‐sheet) like the secondary structure of Aβ, as well as the HDAC6 protein to observe any changes in Aβ deposition and HDAC6 expression with aging. Confocal microscopic images of hippocampus regions were captured in 4X and 40X objectives using Nikon C2 (**Figure** **2**a). Different colored boxes shown in different sub‐regions of brain were further analyzed at higher magnification (40X) and shown in Figure 2b. Merged images revealed overlapping signal of HDAC6 and ThS (shown in white arrows), which suggested the association of higher HDAC6 expression with amyloid deposition in aging that may lead to senile plaques formation in AD. Quantification of ThS‐stained brain images suggested that average sizes and areas covered by the amyloid deposits were significantly higher at different subregions of brain sections in 5‐month‐old 5xFAD mice compared to 3‐month‐old mice (Figure 2c,d). Also, the expression of HDAC6 was significantly higher at dentate gyrus and hippocampus region of 5‐month‐old 5xFAD mice comparing younger mice, whereas the cortex sub‐regions did not show significant change in expression of HDAC6 with aging (Figure 2e). Furthermore, we calculated the number of amyloid and HDAC6‐positive cells in different sub‐areas of brain sections and found significantly higher expression of HDAC6 and amyloid‐positive cells in all the different brain sub‐regions of 5‐month‐old 5xFAD mice (Figure 2f). Hence, small molecule inhibitors of HDAC6 can be a logical approach for developing potential therapeutics for AD. Also, higher expression of HDAC6 and Aβ deposition dependent on age imply the ineffective clearance of Aβ aggregates/plaques by phagocytosis which may be due to impaired microglial activity in aging.

### 2.4 Assessing Effect of PB118 on Murine Macrophages BV2 Cells by Performing Various In Vitro Cellular Analyses

**Figure 3:**
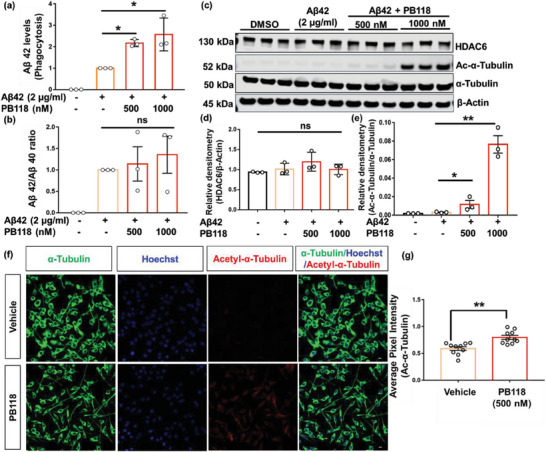
Effect of PB118 on BV2 cells in presence or absence of Aβ42: Different concentrations of PB118 were treated on BV2 cells in presence or absence of Aβ42 and corresponding phagocytosis (a) and ratio of Aβ42/ Aβ40 peptide (b) in cell lysates were measured by MSD‐4G8 kit and quantified using GraphPad Prism software. c) WB immunoassay was performed using PB118 treated cell lysates and probed with HDAC6 protein, acetyl α‐tubulin (Ac‐α‐tubulin), α‐tubulin, and β‐Actin. Quantification of d) HDAC6 expression (normalized with β‐Actin level) and e) Ac‐α‐tubulin expression (normalized with α‐tubulin level) was performed in GraphPad Prism software. f) Immunocytochemistry confocal microscopy images of BV2 cells, were captured in different channels [488, green (α‐tubulin); 405, blue (nuclei); 594, red (acetyl α‐tubulin)] using 40X objective to understand the role of PB118 in restoring the microtubule network in cells. g) Quantification of Ac‐α‐tubulin protein intensity was performed using different images. All the results shown as mean ± SEM (n = 3); Two tailed student's *t*‐test (^*^ *p* <0.05, ^**^ *p*<0.001, ^***^ *p*<0.0001, ns = non‐significant).

The regulation of brain function in health and disease conditions depends on phagocytosis by glial cells, and clearance of Aβ by phagocytosis is probably protective in the early stage of AD but at later stages, impaired phagocytic function of glial cells certainly contributes to poorer disease outcomes. Now, to analyze the effects of our newly developed HDAC6i on microglial phagocytosis regulation, we used the murine microglial BV2 cells and treated with PB118 in the presence or absence of Aβ. Cells were seeded in a 6‐well tissue dish (400k mL^−1^) and cultured overnight for reaching ≈90% confluency. Treatment was performed with vehicle (DMSO), Aβ42 (2 µg mL^−1^), and PB118 (500 and 1000 nm) with or without Aβ (*n* = 3). After 6 h, cell media were collected and the LDH assay was performed that showed no significant cytotoxicity of the PB118 (Figure S4, Supporting Information). This was further validated by capturing microscopic images before and after treatment, which showed no significant change in cell morphology (Figure S4, Supporting Information). Cell lysates were collected after applying MPER++ solution to each well and centrifuging at 12 000 rpm (13 800 × g) for 15 min and further analyzed by the MSD assay using the MSD Aβ peptide panel 1 (4G8) kit to assess the Aβ levels. MSD‐Aβ analysis showed a significant increase in Aβ42 levels in PB118‐treated cell lysates comparing to control (**Figure** **3**a). This result suggested an upregulation of the microglial phagocytosis of Aβ in the presence of PB118 that indicated the possible therapeutic application in AD. We also measured the Aβ42/Aβ40 ratios in the cell lysates but did not find significant changes comparing treatment groups (Figure 3b). Thereafter, WB immunoassay was performed to measure the expression of different protein levels in the PB118‐treated cell lysates. Quantification of the WB membranes with the HDAC6 protein showed non‐significant change in HDAC6 expression, after normalizing to the housekeeping β‐actin comparing the differences among treatment groups. Notably, we found that Ac‐α‐tubulin showed a significant increase, normalized to α‐tubulin, in protein levels of PB118‐treated samples compared to control (Figure 3c–e). Upregulation of Ac‐α‐tubulin is a measure of stable microtubule, which was achieved due to the successful inhibition of HDAC6 protein's activity, i.e., deacetylation of tubulin.

### 2.5 Immunocytochemistry (ICC) and Cold‐Depolymerization Assay to Observe the Effect of PB118 on Intracellular Tubulin/ Microtubule

**Figure 4:**
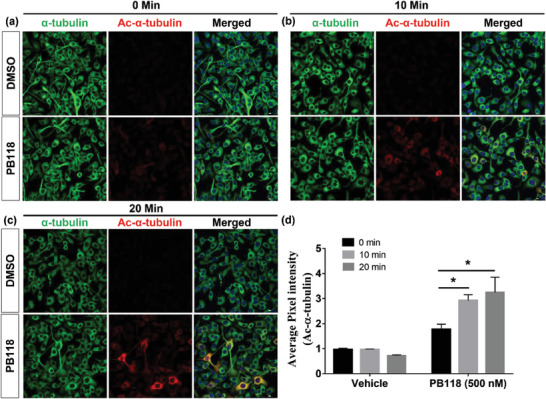
Immunocytochemistry (ICC) study in BV2 cell to observe the effect of PB118 on intracellular tubulin/microtubule in cold depolymerization condition: Cells were treated with DMSO and PB118 (500 nm), fixed with 4% PFA at different time points and performed ICC staining. a–c) Confocal microscopy images of BV2 cells, captured in different channels [488, green (α‐tubulin); 405, blue (nucleus); 594, red (acetyl α‐tubulin)] using 40X objective to reveal the role of PB118 in restoring the microtubule network in cell at cold depolymerization condition. Scale bars corresponded to 10 µm. d) Quantification of acetyl α‐tubulin protein intensity was performed using different images. Results shown as mean ± SEM (n = 3); Student's *t*‐test (^*^ *p* <0.05, ^**^ *p*<0.001, ^***^ *p*<0.0001).

Previous findings suggest significant depolymerization of the tubulin/microtubule network in AD due to the hyperphosphorylation of tau protein that usually stabilizes the microtubule.^[^ ^28^ ^]^ These hyperphosphorylation may be due to the dysfunction of overexpressed HDAC6 in brain cells. So, preventing the activity of HDAC6 by using small molecule inhibitors may result in stabilizing microtubule networks. To further assay this we treated the BV2 cells with PB118 and performed an ICC assay to observe the microtubule network. Primary and secondary antibodies used in this study (**Tables** **1** and **2**) were mixed after fixing the cells with 4% paraformaldehyde (PFA) for 10 min. Confocal microscopy images of BV2 cells were captured in different channels [488, green (α‐tubulin); 405, blue (nucleus); 594, red (acetyl α‐tubulin)] using 40X objective to understand the role of HDAC6is in restoring the microtubule network. Microscopic images reveal that in the presence of PB118, expression of acetyl α‐tubulin is higher compared to vehicle (Figure 3f). Quantification of images showed a significant difference in acetyl α‐tubulin expression in PB118‐treated groups (Figure 3g). So, our newly developed HDAC6i PB118 can significantly increase acetyl α‐tubulin, provide the stability of intracellular microtubule network, and thus may help in combating the neuroinflammation process or AD‐related symptoms. In AD, the severely damaged microtubule network causes several dysfunctions in regular cellular activity. Blocking this rapid disruption of microtubule network in degeneration conditions may successfully delay the neuroinflammation‐related changes in AD. We showed that acetyl α‐tubulin expression was higher when we treated the BV2 cells with PB118. Further, to observe how effective our PB118 is in depolymerizing condition, we performed cold depolymerization assay to evaluate its effect on the stability of tubulin/microtubule morphology at depolymerization condition. Microtubule network gets severely damaged at low temperature as microtubule is only stable at physiological temperature and tubulin polymerization only happens at 37 °C.^[^ ^29^ ^]^ Now, to observe the effect of HDAC6i against cold‐induced microtubule de‐polymerization, PB118‐treated BV2 cells were incubated on ice and fixed at different time points (0, 10, and 20 min) and finally ICC was performed using α‐tubulin (green) and acetylated α‐tubulin (red) primary antibodies. Confocal microscopic images were captured in different channels, merged, and shown in **Figure** **4**a–c. Quantification of the images suggests that acetylated α‐tubulin protein expression was significantly higher in both 10 and 20 min incubated BV2 cells in comparison to 0 min (Figure 4d). These results clearly suggest the effectivity of PB118 even at the depolymerization condition as it increases the level of Ac‐α‐Tubulin, which is a measure of stable microtubule.

**Table 1:** Overview of primary antibodies used in WB of this study.

Primary antibodies	Host	Cat no. & Supplier	Dilution
Acetylated α‐tubulin	Rabbit	5335S, Cell signaling	1:1000
α‐tubulin	Mouse	ab7291, Abcam	1:10000
HDAC6	Rabbit	D21B10, Cell signaling	1:1000
p‐Tau	Mouse	9632S, Cell signaling	1:1000
β‐Actin	Mouse	8H10D10, Cell signaling	1:1000

**Table 2:** Overview of primary/secondary antibodies and dyes used in IHC/ICC of this study.

Primary antibodies and Dyes	Host	Cat no. & Supplier	Dilution
Acetylated α‐tubulin	Rabbit	5335S, Cell signaling	1:1000
α‐tubulin	Mouse	ab7291, Abcam	1:2000
HDAC6	Rabbit	D21B10, Cell signaling	1:500
Thioflavin‐S (ThS)	Dye	T1892, Sigma Aldrich	0.1%
Hoechst	Dye	33342, Thermo Scientific	1 µg mL^−1^
Secondary antibodies			
Anti‐Rat‐A488	Donkey	Jackson Immuno Research	1:500
Anti‐mouse‐A488	Donkey	Jackson Immuno Research	1:500
Anti‐Rabbit‐A594	Donkey	Jackson Immuno Research	1:500
Anti‐mouse‐A640	Donkey	Jackson Immuno Research	1:500

### 2.6 Measuring the Proinflammatory Cytokines after Treating the Lipopolysaccharide (LPS) Induced BV2 cells with Different Concentration PB118

**Figure 5:**
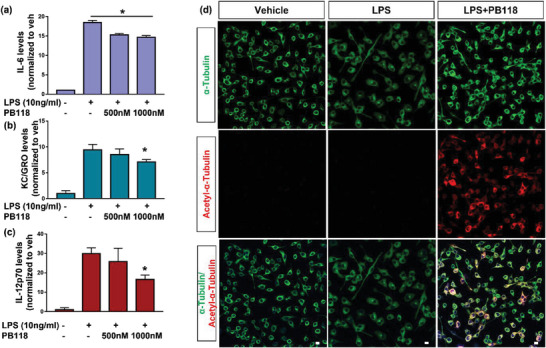
Treatment of PB118 on BV2 cells in presence of LPS to measure the immune protein levels and intracellular tubulin/microtubule: BV2 cells were utilized to assess inflammatory changes induced by LPS. Cells were treated with DMSO, LPS (10 ng mL^−1^), LPS (10 ng mL^−1^) with PB118 (500 nm/1000 nm), for 24 h and the multi‐plex MSD‐cytokine assay was applied to measure levels of cytokines. Change in a) IL6, b) KC/GRO, and c) IL12p‐70 inflammatory cytokines was observed. Mean ± SEM (*n* = 4) Student's *t*‐test (ns = non‐significant, ^*^ *p* <0.05). d) Confocal microscopy images captured in different channels [488, green (tubulin); 594, red (Acetyl‐α‐tubulin)] using 40X objective to understand the effect of LPS in the presence of PB118 in restoring the microtubule network in cell (as HDAC6 is known to disrupt the microtubule network/bundle formation). Scale bar corresponded to 10 µm.

Microglia are the CNS‐resident macrophages that usually respond to brain injury or pathogens by clearing cell debris, misfolded protein aggregates, and damaged neurons by phagocytosis and actively engage with their environment by secreting inflammatory cytokines. The alteration in different inflammatory cytokines levels in the presence of PB118 was measured by the multiplex MSD‐cytokines assay after inducing the cells with lipopolysaccharides (LPS), a well‐characterized inflammatory inducer, and treating with different concentrations of PB118. BV2 cells were treated with 10 ng mL^−1^ LPS alone or in combination with 500 or 1000 nm PB118 for 24 h. Cell media were collected and applied to the LDH assay to assess cell viability and investigate cell tolerance. No significant difference was observed comparing between the treated and control groups (Figure S5, Supporting Information). Stereomicroscopic images, captured before and after treatment of PB118, revealed that there was no significant difference of cell morphology that further confirmed the non‐cytotoxicity nature of this compound (Figure S5, Supporting Information). The multi‐plex MSD‐cytokine assay was performed with PB118‐treated media to measure levels of cytokines and chemokines. Results indicated significant reductions of interleukin‐6 (IL‐6), KC/GRO (CXCL1), and IL‐12p‐70 (**Figure** **5**a–c). IL‐6 is secreted mainly by T‐cells and macrophages that are involved in inflammation, apoptosis, etc., whereas CXCL1 is expressed in macrophages and generates biological signals during inflammation by binding with CXCR2 and involved in neutrophil activation. IL‐12p‐70 activates T‐cells and natural killer cells that stimulate the production of IFN‐γ, which is an activator of macrophages and related neuroinflammation. By reducing these cytokines and chemokines levels, our newly developed PB118 emerges as the potential therapeutic against AD.

### 2.7 Immunocytochemistry to Measure the Change in Intracellular Tubulin/Microtubule or HDAC6 Expression in BV2 Cells After Inducing with LPS

**Figure 6:**
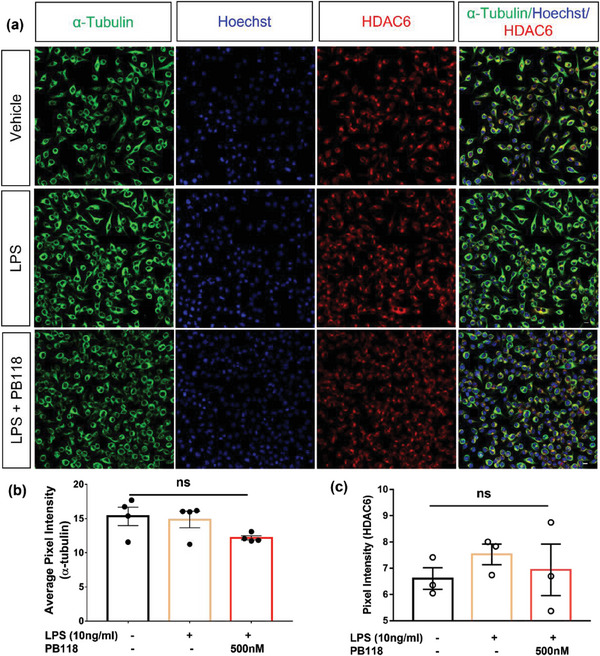
ICC study to observe the effect of HDAC6 in association with intracellular tubulin/microtubule in the presence of LPS: a) Confocal microscopy images of BV2 cells, captured in different channels [488, green (tubulin); 405, blue (nucleus); 594, red (HDAC6)] using 40X objective to understand whether LPS may have any effect on HDAC6 expression or in restoring the microtubule network in cells. Cells were treated with DMSO, LPS (10 ng mL^−1^), LPS (10 ng mL^−1^) with PB118 (500 nm), followed by fixation with 4% PFA for 10 min and incubation with different primary and secondary antibodies, Scale bars corresponded to 10 µm. Quantification of alpha‐tubulin b) HDAC6 protein c) intensity was performed using different images. Results shown as mean ± SEM (*n* = 3); Student's *t*‐test (ns = non‐significant).

Further, ICC‐based analysis was executed to observe the change in intracellular tubulin/microtubule network or HDAC6 proteins expression after inducing the microglia BV2 cells with the neuroinflammation inducer LPS (10 ng mL^−1^) and with PB118 at 500 nM concentration. Following treatment, the cells were fixed with 4% PFA and stained with different primary antibodies; HDAC6, Ac‐α‐tubulin, α‐tubulin, and Hoechst. Confocal microscopy images captured in different channels [488, green (tubulin); 594, red (acetyl‐α‐tubulin)] using 40X objective to understand the effect of LPS in the presence of PB118 in restoring the microtubule network considering that HDAC6 may disrupt the microtubule network/bundle formation. Confocal microscopy images (Figure 5d) clearly showed no change in acetylated tubulin levels in presence of PB118 with or without LPS. In addition, we also checked the HDAC6 expression levels as well after inducing the cells with LPS and treating with PB118. Confocal microscopy images, shown in **Figure** **6**a, indicated lack of significant change in α‐tubulin network or HDAC6 expression in BV2 cells after treating with LPS (10 ng mL^−1^) and PB118, which was further confirmed by quantifying by the Image J (Figure 6b,c). Collectively, these data support not only that HDAC6 is a key player reducing acetyl‐α‐tubulin and altering microtubule network in microglia, as expected, but also provide new evidence of our new small molecule inhibitor of HDAC6 PB118 in improving acetyl‐α‐tubulin and microtubule network.

### 2.8 3D Human Neural Stem Cell Differentiation and Treatment with PB118

**Figure 7:**
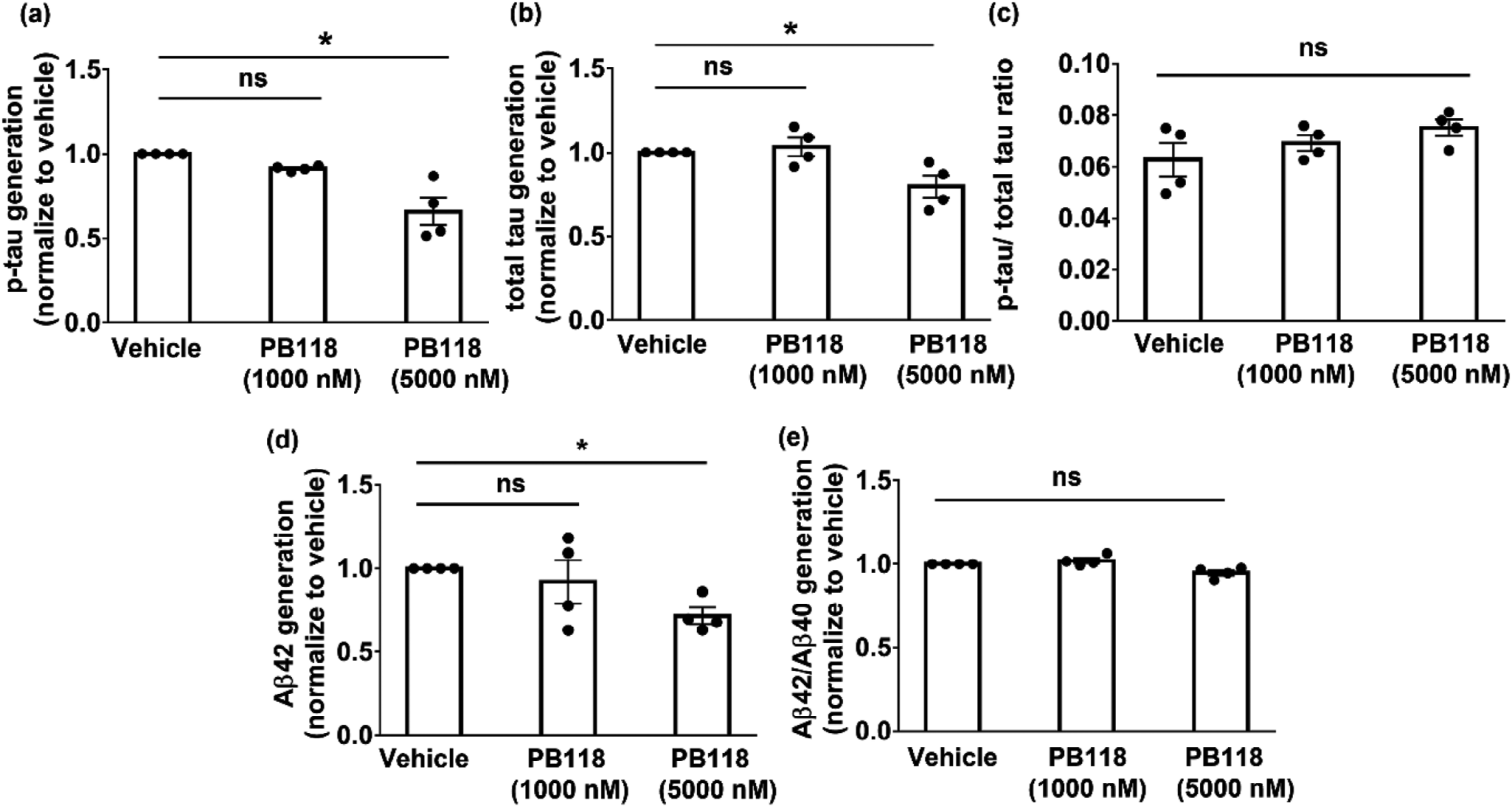
MSD analysis to measure the p‐tau and Aβ generation after treating 3D human neural stem cell with PB118: 3D cells were treated with PB118 and a) p‐tau, b) total tau, c) p‐tau/total tau, d) Aβ42 generation and e) Aβ42/Aβ40 ratio were measured by MSD assay. Quantification of results was performed using Student's *t*‐test (^*^ *p* <0.05, ^**^ *p* <0.001, ns = non‐significant). Results shown as mean ± SEM (n = 3).

Recently, a 3D human neural stem cell model of AD using APP and presenilin‐1 overexpressing ReNcell VM human neural stem cells was developed that can express both amyloid and tau pathology with high levels of phosphorylated tau (phospho‐tau; p‐tau) in the system. The research on 3D‐AD human neural culture model is gaining more interest, making it a particularly valuable tool system to analyze AD‐related tau pathology in drug discovery.^[^ ^30^ ^]^ Thus, we used this 3D‐AD culture model to measure the change in Aβ and p‐tau levels in the system after treating with different concentrations of PB118 to evaluate its anti‐AD effectivity. Wild type (G10), and APP mutant (A5) 3D‐plated cells were cultured and differentiated for 6 weeks followed by the treatment with 1 or 5 µm of PB118 and vehicle (DMSO) and maintained for an additional 3 weeks. Cell lysates were collected, analyzed by the MSD Phospho (Thr 231)/total tau and MSD Aβ (6E10) assay kit and the LDH assay for accessing p‐tau and Aβ levels and cytotoxicity respectively. The LDH assay showed no cytotoxicity of PB118 on 3D human neural culture (Figure S6, Supporting Information) whereas MSD analysis showed that higher concentration of PB118 significantly reduced p‐tau and total tau levels in the system (**Figure** **7**a,b) though the ratio of p‐tau/total tau was not changed (Figure 7c). These results suggest therapeutic application of PB118 as it reduces the p‐tau generation, which blocks neurofibrillary tangles (NFTs) formation in AD. Also, Aβ42 generation in the system was significantly reduced as a function of PB118 and though the ratio of Aβ42/Aβ40 did not change that suggests further degradation of amyloid protein in the presence of PB118 (Figure 7d,e). Collectively, these data support the multi‐modal mechanisms of PB118 in reducing AD pathology, including amyloid pathology by increasing Aβ uptake and reducing Aβ generation, reducing tau phosphorylation, improving acetyl‐α‐tubulin and microtubule network, as well as reducing inflammatory proteins closely associated with AD. These results suggested that the anti‐AD mechanism of PB118 in blocking p‐tau related NFTs formation and reducing Aβ generation, the two main pathological changes of AD.

### 2.9 Effect of PB118 on Human Microglia (HMC3) Cells

In this work, we have seen that PB118 does not have any toxicity on murine microglia BV2 cell line, 3D human neural stem cell but showed its intent to be a potent therapeutic in AD by effectively reducing various AD neuropathological hallmarks. Further to access the biocompatibility of PB118, we treated the human microglia HMC3 cells with PB118. Stereomicroscopic images of before and after treatment (Figure S7, Supporting Information) with vehicle and PB118 followed by LDH assay (Figure S7, Supporting Information) indicated no detrimental change in morphology or cytotoxicity of PB118. These results further validated the biocompatibility and future application of PB118 in AD therapeutics.

## 3 Conclusion

In summary, the results of our current study provided strong evidence supporting the structure‐based drug design of potent and specific small molecule inhibitors of HDAC6 with compelling efficacy in attenuating multiple neuropathology of AD in disease models. Our study was able to provide new insight on the pathophysiology of HDAC6 underlying AD.

HDAC6 has been a well‐known key player involved in a wide range of biological functions, such as clearance and degradation of aggregated proteins, regulation of immunological responses, and neurological pathogenesis. Dysfunction of HDAC6 closely associated with disease outcome and targeting HDAC6 as anti‐cancer therapeutics has been extensively investigated with some potential drugs being advanced in clinical trials.^[^ ^13^ ^]^ Nevertheless, the relevance of HDAC6 to the pathogenesis of AD and its potential as a drug target for AD is still unclear and needs in‐detail investigation for developing therapeutics. Our previous finding showed that HDAC6 is highly expressed in AD brains and may be responsible for pathological changes leading to AD. In this work, we showed that the amyloid deposition and HDAC6 expression is higher in aged AD mice which encouraged us to further investigate mechanism of actions of our new small molecule inhibitor PB118 underlying AD. We rationally developed this HDAC6i PB118 that binds with HDAC6 enzymes with >100‐fold selectivity compared to other histone deacetylases. Consistent with the biological functions of HDAC6 in protein aggregation, PB118 significantly impacted the aggregation‐prone Aβ42 protein and increased its phagocytosis, a process for clearing cell debris and protein aggregates in microglia BV2 cell. Along with this, PB118 significantly increased the acetylated α‐tubulin levels in BV2 cell, which is a measure of stable microtubule, by inhibiting the activity of HDAC6. Further, the cold depolymerization assay revealed that PB118 can provide sufficient stability at the depolymerizing condition induced by cold environment that proved the potential robust therapeutic application of PB118. We also checked PB118 activity in presence of LPS, to assay the neuroinflammation‐related cytokines and chemokines changes in microglia. This inhibitor can successfully reduced the IL‐6, IL‐12p‐70, and KC/GRO (CXCL1) cytokines and chemokines which are responsible for neuroinflammatory changes in AD. Finally, we assessed the effects of PB118 on 3D‐AD human neural culture model, and found significant reductions of p‐tau levels and Aβ42 generation. Collectively, our studies supported the multi‐modal mechanisms of action of our new HDAC6i PB118 in attenuating AD pathology, including reducing amyloid pathology by increasing Aβ uptake and lowering Aβ generation, reducing tau phosphorylation, improving Ac‐α‐tubulin and microtubule network, as well as reducing inflammatory events associated with AD. Thus, our data on pharmacophore analysis and structure‐based designing of HDAC6is in combination with mechanisms of action findings using disease models not only suggests PB118 as a clinically promising molecule, but also supports the significance of further investigation on HDAC6 and its inhibitors which may be advanced toward future translational studies.

## 4 Experimental Section

### Reagents, Media, and Cell Lines

All the chemicals used in this study are commercially purchased and used without further purification. Lipofectamine (LPS) was purchased from Thermo Fisher (00‐4976‐93); Beta Amyloid (1–42) was procured from Anaspec (AS24224), Mammalian protein extraction reagent (MPER; #78 505)), EDTA, Protease inhibitor (78 425) were purchased from Thermo Fischer Scientific. For 2D cell culture, murine microglia BV2 cell was used, human microglia HMC3 and cultured in Dulbecco's modified Eagle's media (12‐707F; Lonza) supplemented with 10% fetal bovine serum (FBS), L‐Glutamine (25‐005‐Cl; Corning), and Penicillin/streptomycin (DE17‐602E; Lonza). Cells were cultured in a 37 °C incubator with water‐saturated air and a 5% CO_2_ atmosphere and splitting was done every 2–3 days using trypsin (BE17‐161E; Lonza).

### Pharmacophore Analysis and Molecular Docking with HDAC6 and PB118

Pharmacophore analysis was performed using some already published HDAC6is that reveal of three main parts: surface surface‐binding capping group, a linker, and a zinc‐binding group. A hydroxamate group was introduced for binding Zn^+2^ and several rigid diphenyl groups for better binding of the inhibitors with HDAC6 enzyme. Different inhibitors were synthesized using various capping groups and evaluated their activity by the HDACs enzyme inhibition assay and reported in the previous work. This study found that PB118 had the most selectivity toward HDAC6 enzymes and therefore the molecular docking was performed to evaluate the binding analysis of PB118 in HDAC6. For that, the PDB structure of HDAC6 (6THV)^[^ ^26^ ^]^ and PB118 (PDB structure was generated by AVOGADRO^[^ ^31^ ^]^) were used, and docking was performed in the online server HDOCK.^[^ ^25^ ^]^ Molecular docking images were visualized and developed using UCSF Chimera.^[^ ^32^ ^]^

### Synthesis of HDAC6 Inhibitor PB118

Detailed synthesis procedure, mass, and NMR results for all the intermediates and final PB118 compound were reported in the previous manuscript.^[^ ^15^ ^]^ A schematic representation of the synthetic route and NMR results are shown in Figure S1 (Supporting Information). Briefly, to a stirred mixture of 2,3,4,5‐tetrahydro‐1*H*‐benzo[b]azepine (100 mg, 0.68 mmol) and K_2_CO_3_ (187.9 mg, 1.36 mmol) in acetone (10 mL), and methyl 4‐(bromomethyl)−3‐fluorobenzoate (209.4 mg, 0.68 mmol) was added, and this mixture was further stirred at 60 °C for 6–8 h. After the completion of reaction, the acetone was evaporated under reduced pressure and the residue was purified by silica gel chromatography to afford the ester intermediate. The easter intermediate (0.25 mmol,) was dissolved in THF/MeOH (1:1, 1.0 mL) and then added to a stirred solution of solid NaOH (40 mg, 1.0 mmol) in 50% aqueous NH_2_OH (0.8 mL) dropwise at 0 °C (Figure S1, Supporting Information). The resulting mixture was further stirred for another 30 min while warming to room temperature. The solution was neutralized with 2n HCl to pH ≈7. The crude products were purified by a reverse phase C‐18 column using Combiflash and lyophilized to give PB118 as a white powder.

### Stability of PB118 in Physiological Condition^[^ ^33^ ^]^

To evaluate the suitability of PB118 in in vivo system, a stability experiment was performed with PB118 in physiological conditions. PB118 (5 mm) was mixed with human serum and kept at continuous stirring at 37°C. High‐pressure liquid chromatography (HPLC) was used to measure the intensity of PB118 after injecting 100 µL of the mixture solution at different time points (0, 1, 2, 4, 6, 12, and 24 h) and monitored for 24 h. HPLC results were recorded and a graph was prepared using GraphPad Prism and half‐life (t_1/2_) of PB118 in human serum was calculated following the equation in Figure S2 (Supporting Information).

### Enzyme (HDAC1‐11) Inhibition Assay with PB118

The HDAC enzyme assays were performed and reported in the previous publication.^[^ ^15^ ^]^

In brief, HDAC inhibition assay of target compounds was carried out at Nanosyn (Santa Clara, CA, USA). Test compounds were diluted in 100% DMSO using threefold dilution steps. The final compound concentration in the assay ranged from 10 mmol/L to 0.056 nmol L^−1^. Compounds were tested in a single well for each dilution, and the final concentration of DMSO in all assays was kept at 1%. Reference compounds, TSA was tested in an identical manner.

### Microglial BV2 Cell‐Based Experiments

This study utilized microglial BV2 cells and tested the newly developed PB118 to see whether it could reduce microglia‐related neuroinflammation using lipopolysaccharide (LPS), a well‐characterized inflammatory inducer. Briefly, for microglia‐related amyloid (Aβ) phagocytosis study, BV2 cells were seeded in a 6‐well confocal dish (400k cells mL^−1^) and cultured overnight for ≈90% confluency. Then, cells were treated with DMSO (vehicle) and Aβ (2 µg mL^−1^) alone or in combination with 500 and 1000 nm of PB118 in serum‐free complete media for 6 h. Then, media were collected to perform the LDH assay for cell cytotoxicity assessment. Cells were washed carefully with PBS (2x) and lysed by adding 200 µL of MPER++ (10 mL MPER with 1% protease cocktail inhibitor and 1% EDTA) in each well. Cell lysates were collected in 1.5 mL Eppendorf tube and centrifuged at 12 000 rpm at 4 °C for 15 min and supernatants were collected for further studies. Total protein was quantified using the BCA protein assay kit (Pierce). Briefly, BV2 cells were cultured following previously published methods and grew in DMEM media containing 10% heat‐inactivated fetal bovine serum, 2 mm l‐Glutamine, and 1% penicillin/streptomycin (Life Technologies). BV2 cells were treated with DMSO (Vehicle), 10 ng mL^−1^ LPS alone or in combination with 500 and 1000 nm of PB118 in serum‐free media for 24 h. Subsequently, media were collected and applied to LDH to assess cell viability; cells were extensively washed with PBS and were lysed in MPER lysis buffer (MPER++) supplemented with EDTA‐free protease inhibitors (Roche), Halt phosphatase inhibitor cocktail (Thermo Fisher Scientific). Lysates were centrifuged as before (12 000 rpm at 4 °C for 15 min) and supernatants were collected for further analysis.

### 3D Cell Cultures and Differentiation

Human precursor ReN cells were used to prepare 3D AD environment following previous work.^[^ ^30^ ^]^ Briefly, for 3D layers of cells, BD Matrigel stock solution (BD Biosciences) was diluted with ReN cell differentiation medium (in 1:15 dilution) (chilled) followed by vertexing with the cell pellets for 20 s. A final cell concentration of ≈2 × 10^6^ cells mL^−1^ was used and this cell/ Matrigel mixtures were immediately transferred (100 mL in each) into an Optilux clear bottom 96‐well plates (BD Biosciences). A thin layer (100–300 mm) of 3D gels was formed at the bottom of the plates after incubating the plates at 37 °C for 1 h and the media were changed. For differentiation before drug treatment, 3D‐plated cells were cultured for 6 weeks, and the media were changed every 3–4 days. For drug treatments, differentiation media containing with 1 and 5 μm of PB118 and DMSO (1 µL in 1 mL) were added to 6‐week differentiated 3D‐cultured ReN cells and then maintained for an additional 3 weeks. The cells were harvested for extraction using a lysis buffer mixture (Lysis 6 buffer with protease inhibitor; 1:100), followed by shaking for 1 h at cold room and centrifugation at 2 k rpm for 30 min. Supernatants were collected and further analyzed by MSD immunoassay kit and western blot analysis.

### Lactate Dehydrogenase (LDH) Assay

The previously reported CytoTox‐ONE assay was performed to analyze cell viability by measuring the release of lactate dehydrogenase (LDH) following the manufacturer's guidelines (Promega; Madison, WI). Briefly, 50 µL of cell culture media was utilized and a 1:1 dilution of substrate was added. The plate was incubated for 30 min in a 37 °C incubator with the signal measured using a spectrophotometer at an excitation wavelength of 560 nm. Under the influence of the assay's substrate, resazurin was converted to resorufin, its fluorescent form, owing to LDH. The fluorometric results were used to compare the experiment group to the control group to assess the cytotoxicity of treated compounds.

### Stereomicroscopic Images Before and After Treatment on BV2 Cells

Stereomicroscopic images were captured before and after the treatment of different concentrations of PB118 on BV2 cells to elucidate the cytotoxicity based on the cellular morphology of BV2 cells by using Invitrogen EVOS XL Core stereo microscope.

### Western Blotting (WB) Analysis

The previously described WB method was implemented to perform and analyze western blot membrane.^[^ ^34^ ^]^ Briefly, PB118‐treated BV2 cell lysates were collected, protein concentrations were measured using the Pierce BCA protein kit (Pierce 23 225) and an equal amount of proteins were applied to the Wb analysis. Proteins were separated by the electrophoresis using the 4–12% Bis‐Tris SDS‐PAGE Gel System (Invitrogen, Thermo Fisher Scientific), followed by membrane transfer to PVDF membranes (Invitrogen, Thermo Fischer scientific). Next, the membranes were blocked for 30 min with the Superblock (Thermo Scientific) and then incubated overnight with primary antibodies following the dilutions mentioned below. The membranes were then washed with TBST (four times) and incubated with appropriate secondary antibodies (1:10 000 dilution) at room temperature for 2 h. Then, membranes were incubated with Supersignal West Femto (Life Technologies), and the images for immunoreactivity were developed and visualized by the LICOR Odyssey Fc. Quantifications of proteins of interest were performed by Image J software.

### MSD Analysis (Aβ, Inflammatory Cytokines)^[^ ^34^, ^35^ ^]^

MSD immunoassays are quite robust and based on combining multiarray technology and electrochemiluminescence to identify numerous proteins in a single sample. To evaluate the Aβ phagocytosis, BV2 cells were treated with Aβ (2 µg mL^−1^) and PB118 (500 and 1000 nm) compounds to measure the change in Aβ levels in cells in the presence of PB118 by performing mesoscale discovery (MSD) immunoassays. Also, the change in different inflammatory cytokines levels was measured by multiplex MSD‐cytokines assay after inducing the cells with lipopolysaccharides (LPS) and treating with different concentrations of PB118. MSD Aβ peptide panel 1 (4G8) and MSD proinflammatory panel 1 (mouse) kit was used for measuring the protein levels (Aβ or different cytokines) in BV2 cell treated with vehicle, LPS or PB118 in present or absence of LPS. MSD Aβ peptide panel 1 (6E10) and MSD phospho (Thr231)/total tau kit were used to evaluate the amount of Aβ or phospho‐tau/total present in the 3D cell lysates after treating the 3D cells with different concentrations of PB118.

### Immunohistochemistry (IHC) Study

IHC study was performed following previously well‐documented works^[^ ^15^ ^]^ using brain sections of male 5xFAD animals with approvals granted by theInstitutional Animal Care and Use Committee at Massachusetts General Hospital. Briefly, the brains were first incubated in 4% paraformaldehyde at 4 °C for 28 h, and then for cryoprotection, these were incubated in sucrose (30%) solution in PBS. Coronal brain sections were dissected in 40 µm slices using a sliding microtome. Then, these free‐floating brain sections were washed with PBS (3X) for 10 min, then blocked with blocking buffer (5% Donkey serum with 0.1% Triton‐X‐100 in PBS) for 1 h followed by overnight incubation with appropriate primary antibody following the table listed below. For negative staining, instead of a primary antibody, an equal amount of blocking buffer was added. Then, these brain sections were washed with plenty of PBS followed by incubation with appropriate secondary antibodies for 2 h at rt and washing three times with PBS. If ThS staining was required, then these brain sections were incubated with 0.1% ThS solution in PBS for another 10 min followed by washing with PBS until the solution became clear. Brain sections were incubated with 2 nm of Hoechst solution for 10 min followed by PBS washing. Finally, these brain sections were mounted in a glass slide with Prolong Gold antifade reagent, dried at r.t., covered with a cover slip, and imaged with Nikon C2 confocal microscopy. Negative staining was performed to validate the non‐specific staining of 2° antibody. Images shown in Figure S8 (Supporting Information) indicate no such nonspecific staining of 2° antibodies used in this study.

### Immunocytochemistry (ICC) Study on PB118‐ Treated BV2 cells

ICC was performed following previously published articles.^[^ ^36^ ^]^ Briefly, BV2 cells were seeded and grown overnight in a LabTek 8‐well chamber wells having ≈100 k cells well^−1^. Carefully, washed the cells with PBS (2X) at room temperature and treated with DMSO, LPS (10 ng mL^−1^), Aβ (2 µg mL^−1^), and PB118 (n = 2) at 500 nm concentration for 3 h in serum‐free complete media with or without LPS (10 ng mL^−1^)/Aβ (2 µg mL^−1^). Discarded the media after 3 h, washed each well carefully with 150–300 µL of PBS (2x), and fixed with 4% PFA for 20 min at r.t. After that, washed each well again with 150–300 µL of PBS (2x) at r.t and incubated with blocking buffer (5% Donkey serum with 0.1% Triton‐X‐100 in PBS). Aspirate the blocking buffer and add 150 µL of primary antibody in each well and keep it at 4 °C for overnight shaking. Washed with washing buffer (PBS containing 0.1% Triton‐X‐100, 0.05% Tween‐20) twice and incubated with specific secondary antibody in each of the wells with gently shaking for 2 h at r.t. Discarded secondary solution and washed with washing buffer twice and incubated 10 min with 2 nm of Hoechst solution for another 10 min at r.t, washed with PBS (two times) and finally mounted with Prolong Gold antifade reagent, dried at r.t, and covered with a cover slip. Microscopic images were captured with these slides to understand the activity of HDAC6 toward tubulin/microtubule.

### Cold Depolymerization Assay^[^ ^37^ ^]^

To perform this assay, BV2 cells were seeded and cultured in an 8‐well Labtek chamber slide for overnight (100 k well^−1^). These cells were treated with vehicle (DMSO), and PB118 (500 nm) for 24 h. Then cells were incubated on ice to induce microtubule depolymerization and were fixed using 4% PFA at different time points (0, 10, and 20 min). After that, cells were washed with PBS three times and blocked with blocking buffer (5% Donkey serum with 0.1% Triton‐X‐100 in PBS) and immunostaining was performed with these fixed cells against α‐tubulin (green) and acetylated α‐tubulin (red) antibodies. Appropriate secondary antibodies were used for visualization of these cells and images were captured in a Nikon C2 confocal microscopy.

### Effect of PB118 on Human Microglial HMC3 Cell

PB118 was tested on murine microglia BV2 as well as 3D human neural stem cells and had seen no detrimental effect of PB118. Even PB118 was quite stable in human serum and crosses the BBB when intraperitoneal injection was administered on C57BL/6 mice. Further to assess the biocompatibility of PB118, the human microglial HMC3 cells were treated with PB118 (500 and 1000 nm) for 6 h. Stereomicroscope images were captured before and after treatment and LDH assay (detailed method described earlier) was performed with the cell media to evaluate the toxicity of PB118 on HMC3 cells.

### Statistical Analysis

Confocal microscopic images were processed and analyzed using Image J software. WB images for immunoreactivity were developed and visualized by the LICOR Odyssey Fc and proteins of interest were analyzed using Image J software. All the results for different experiments in this work were shown as mean ± SEM. Statistical significances of different results were performed using two tailed Student's *t*‐test in GraphPad PRISM. Values were considered as significant when it comes *p* <0.05.

## Conflict of Interest

The authors declare no conflict of interest.

## Supporting information

Supporting Information

## Data Availability

The data that support the findings of this study are available in the supplementary material of this article.
