# Supplementary material for: Structure‐Based Discovery of A Small Molecule Inhibitor of Histone Deacetylase 6 (HDAC6) that Significantly Reduces Alzheimer's Disease Neuropathology
Source: Adv Sci (Weinh). 2023 Nov 21;11(1):2304545. doi: 10.1002/advs.202304545 (PMC10767396; doi:10.1002/advs.202304545)
Supplement: Supplementary file 1 — Supporting Information [file ADVS-11-2304545-s001.pdf]

ADVANCED SCIENCE
Open Access

Supporting Information

for *Adv. Sci.*, DOI 10.1002/advs.202304545

Structure-Based Discovery of A Small Molecule Inhibitor of Histone Deacetylase 6 (HDAC6) that Significantly Reduces Alzheimer's Disease Neuropathology

Prasenjit Mondal, Ping Bai, Ashley Gomm, Grisilda Bakiasi, Chih-Chung Jerry Lin, Yanli Wang, Se Hoon Choi, Rudolph E. Tanzi, Changning Wang and Can Zhang**

Supporting Information

Structure-based discovery of small molecule inhibitor of histone deacetylase 6 (HDAC6) that significantly reduces Alzheimer's disease neuropathology

Prasenjit Mondal[a], Ping Bai[b], Ashley Gomm[a], Grisilda Bakiasi, Chih-Chung Lin, Yanli Wang,[b] Se Hoon Choi[a], Rudolph E. Tanzi[a], Changning Wang[b,], Can Zhang[a,]**

[a]*Genetics and Aging Research Unit, McCance Center for Brain Health, MassGeneral Institute for Neurodegenerative Disease, Department of Neurology, Massachusetts General Hospital, Harvard Medical School, Charlestown, MA 02129, USA*

[b]*Athinoula A. Martinos Center for Biomedical Imaging, Department of Radiology, Massachusetts General Hospital, Harvard Medical School, Charlestown, MA 02129, USA*

*Corresponding authors
Email: CWANG15@mgh.harvard.edu
Email: zhang.can@mgh.harvard.edu

Supporting Figures:

Step 1

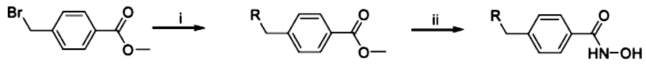

Compound	R=	Compound	R=
12a	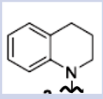	12f	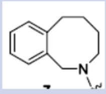
12b	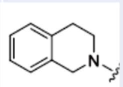	12g	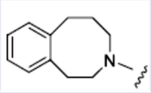
12c	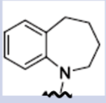	12h	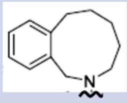
12d	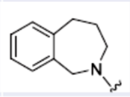	12i	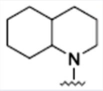
12e	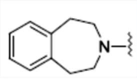		

Step 2

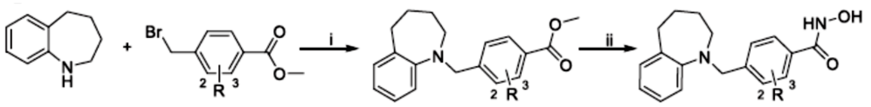

Compound	R=	Compound	R=
15a	2-OCH_3	15c	2-F
15b	3-OCH_3	15d	3-F

Figure S1: Schematic representation of N-heterobicyclic HDAC6 inhibitors synthesis; Reagents and conditions: (i) K_2CO_3, acetone reflux for 6-8h; (ii) NaOH, 50% (w/w), aq. NH2OH, (1:1) THF/MeOH, O °C to rt, for 1 h. (Reprinted with permission from Bai et al, Acta Pharm. Sin. B. 2022, 12(10), 3891, Ref 15, Copyright with Elsevier)

Mass and NMR results for PB118

3-Fluoro-N-hydroxy-4-((2,3,4,5-tetrahydro-1H-benzo[b]azepin-1-yl)methyl)benzamide (**15c, PB118**). Yield: 47.5%. ^{1}H NMR (500 MHz, DMSO-d6) d 9.36 (s, 1H), 7.47 (d, J Z 7.9 Hz, 1H), 7.40 (d, J Z 11.6 Hz, 1H), 7.33 (td, J Z 7.8, 2.1 Hz, 1H), 7.07e7.00 (m, 2H), 6.91 (d, J Z 7.8 Hz, 1H), 6.77 (td, J Z 7.4, 2.1 Hz, 1H), 4.29 (d, J Z 2.2 Hz, 2H), 2.87e2.80 (m, 2H), 2.77e2.70 (m, 2H), 1.51 (q, J Z 9.1, 7.2 Hz, 4H). ^{13}C NMR (126 MHz, DMSO-d6) d 152.1, 135.9, 130.3, 130.0, 127.1, 122.1, 121.5, 117.9, 112.9 (2C), 54.1, 51.1, 40.0, 34.7, 30.0, 25.8. MS (ESIþ): 315.2 [M þ H]þ. 19F NMR (282 MHz, DMSO-d_6): d _119.6 (s, 1F). HRMS (ESI): exact mass calculated for $C_{18}H_{19}FN_2O_2$ [M^+ H^+], 315.1531; found, 315.15046. (Reproduced with permission from Bai et al, Acta Pharm. Sin. B. 2022, 12(10), 3891, Ref 15, Copyright with Elsevier)

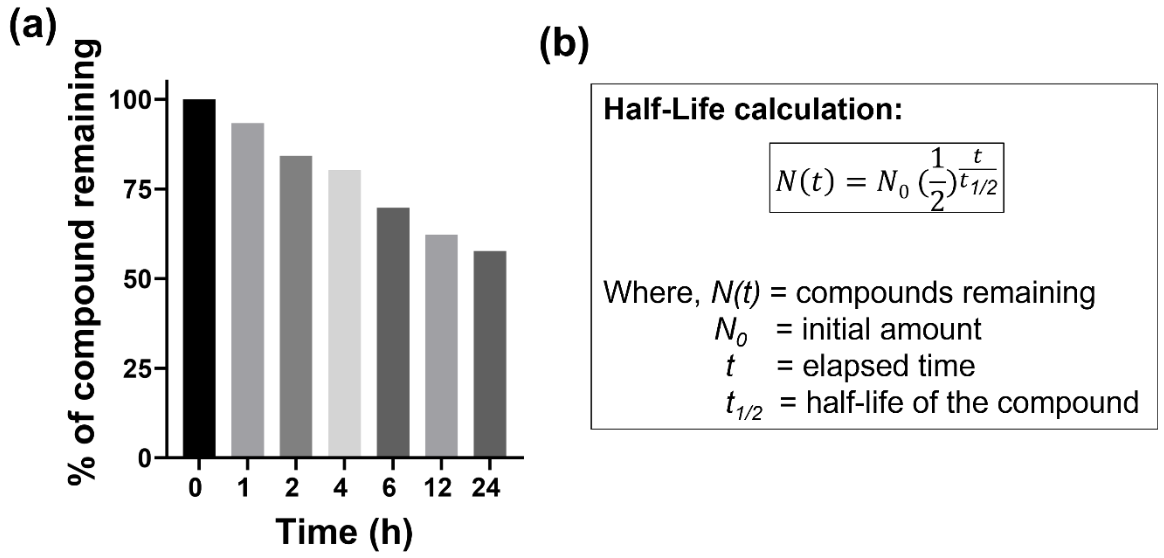

Figure S2: Robust stability of PB118 in human serum; (a) Stability of PB118 compound in human serum was monitored for 24 h and measured using reverse phase High-Pressure Liquid Chromatography (HPLC). (b) Half- life ($t_{1/2}$) of PB118 was calculated using this equation, which is ~30.2 h.

ADMET profile (In vitro)			Brain/plasma PK studies (In vivo; ip 1mg/kg)			
			**Time**	**30 min**	**1 hr**	**4 hr**
**Liver microsomal stability ($t_{1/2}$, min)**	Human	67.9	**Conc. in brain (ng/ml)**	48.5	11.5	0.82
**Hepatocyte stability ($t_{1/2}$, min)**	Human	32.4	**Conc. in plasma (ng/ml)**	1.32	0.72	0.35
**Plasma protein binding (%)**	Human	99.4%	**Brain/plasma ratio**	37	16	2.3

Figure S3: ADME/PK studies with PB118; HD Biosciences Co., Ltd performed the ADME studies and *in vivo* PK profiling. C57BL/6 mice were administered with 1mg/kg PB118 (left). For brain /plasma study, it was followed by the collection of blood and brain samples at 30 min, 1 hr, and 4 hr time points. Samples were processed using acetonitrile precipitation and analyzed by LC-MS/MS (right). (Reprinted with permission from Bai et al, Acta Pharm. Sin. B. 2022, 12(10), 3891, Ref 15, Copyright with Elsevier)

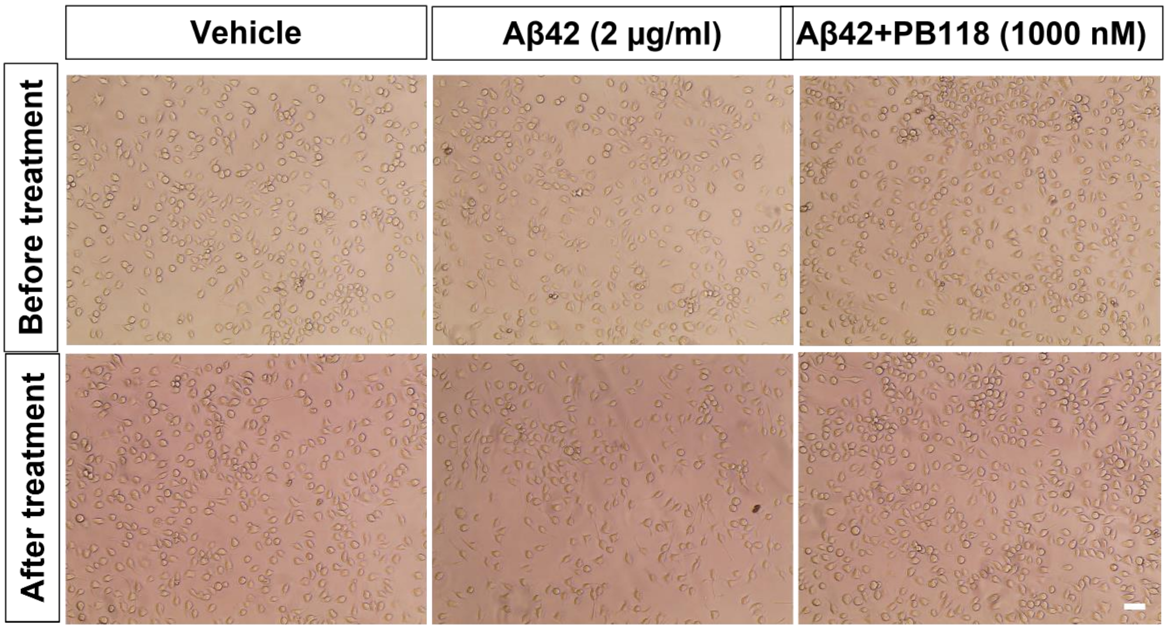

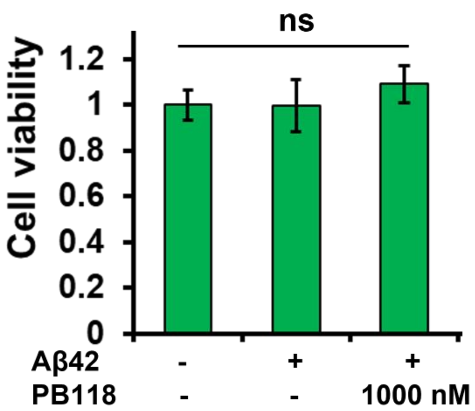

Figure S4: Stereomicroscopic images of mouse microglia BV2 cells before and after treatment with Vehicle (DMSO), Aβ42 (2 µg/ml), and Aβ42 with PB118 (1000 nM) and cell viability assay was tested by the LDH assay. Stereo microscopic images of BV2 cells before and after (a) treatment with PB118 in presence or absence of Aβ42. (b) Cell toxicity of PB118 was analyzed by the LDH assay. Results represented as mean ± SEM; n = 3; No significance difference was observed after analyzing Student's t-test ($p>0.05$). Scale bar corresponded to 100 µm.

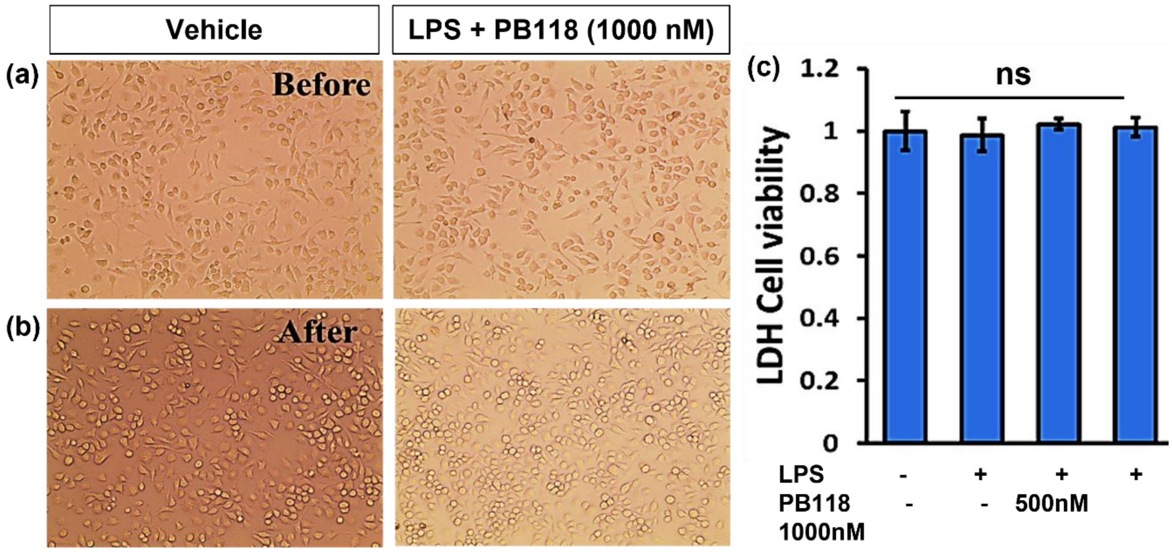

Figure S5: Stereomicroscopic images of mouse microglia BV2 cells before and after treatment with PB118 and cell viability assay by LDH assay. Representative stereo microscopic images before (a) and after (b) treatment with PB118. (c) Cell viability was analyzed by the LDH assay for cells treated with LPS +/- PB118 (500 nM and 1000 nM). Mean ± SEM; n = 3; No significant difference was observed after analyzing Student's t-test (p=0.768). Scale bar corresponded to 100 µm.

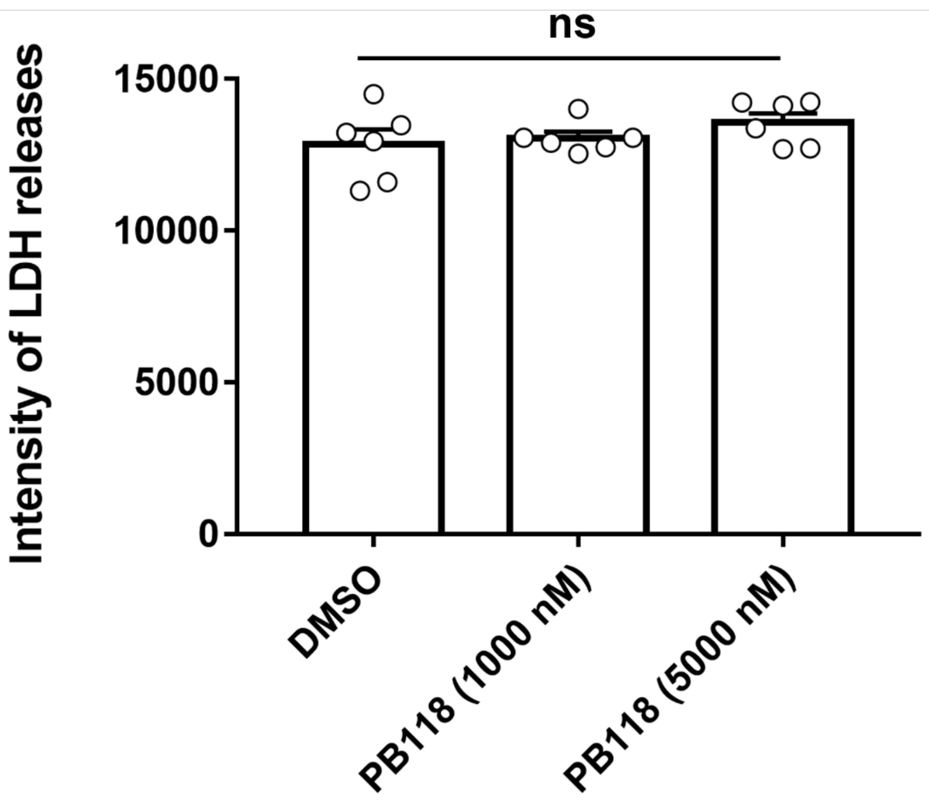

Figure S6: LDH assay to measure the 3D cell viability after treating with PB118. 3D cell viability was analyzed by the LDH assay after cells were treated with PB118 at various concentrations (0, 1000 nM and 5000 nM). Mean ± SEM; n = 3; significance was measured after analyzing two-tailed Student's t-test (ns= non-significant).

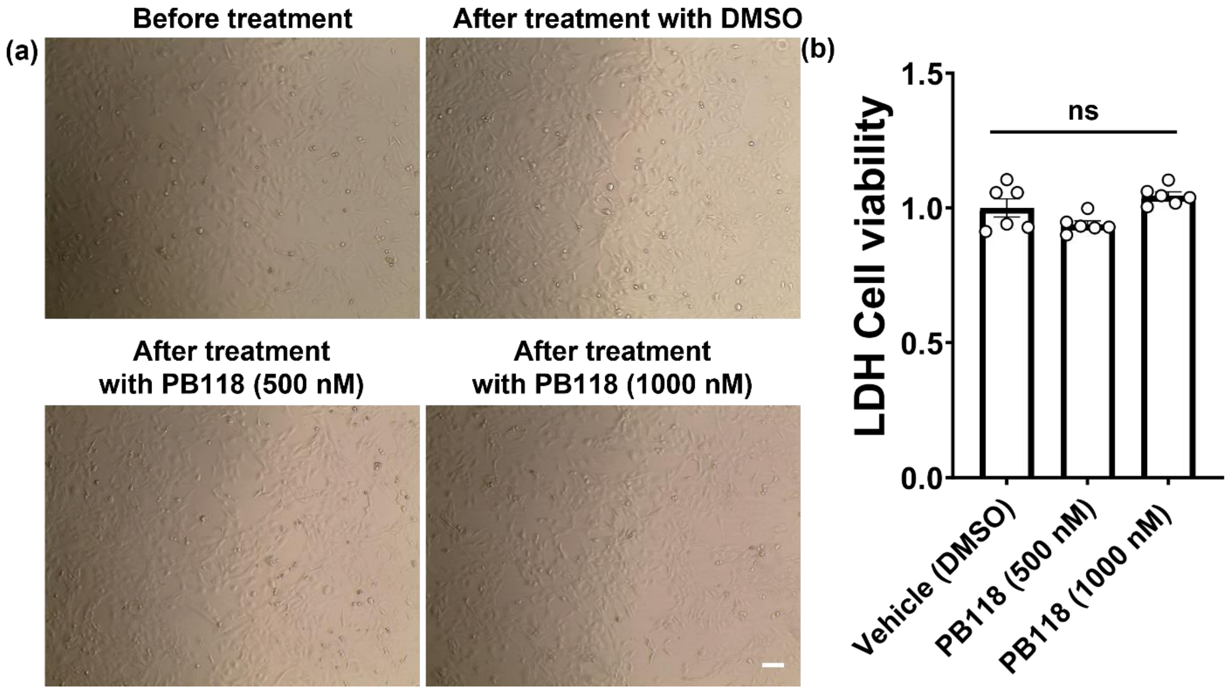

Figure S7: LDH assay to measure the effect of PB118 on HMC3 cells. Cell viability was analyzed by the LDH assay after HMC3 cells were treated with PB118 at various concentrations (0, 500 nM and 1000 nM). Mean ± SEM; n = 3; statistical significance was measured after analyzing two-tailed student's t-test (ns=non-significant). Scale bar corresponded to 100 µm.

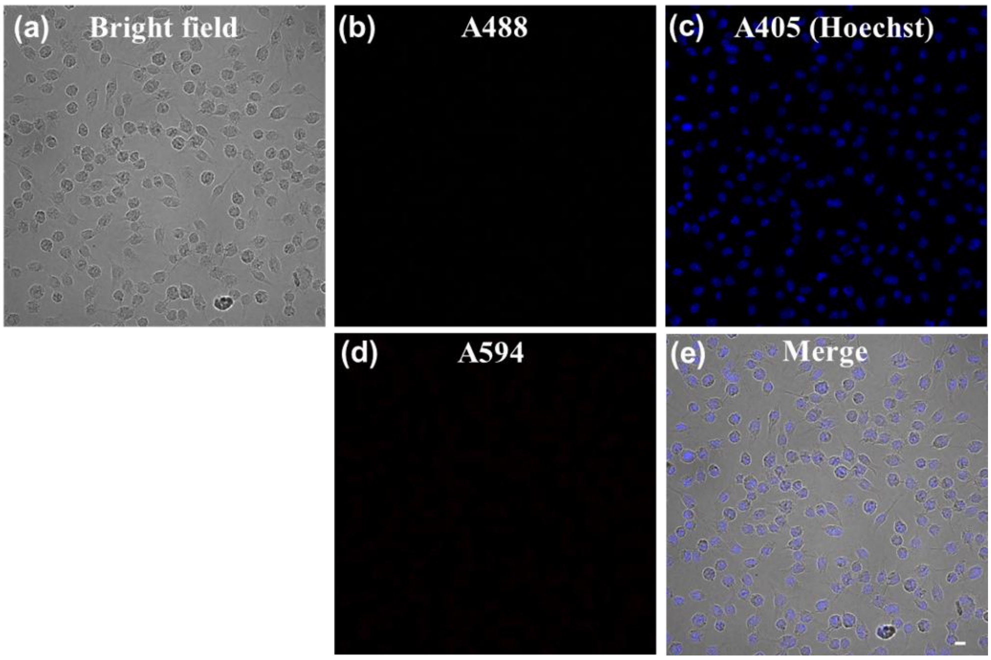

Figure S8: Negative staining confocal images of BV2 cells stained with only secondary antibody (in the absence of primary antibody); Donkey Anti-mouse Alexa 488, Donkey Anti-rabbit Alexa 594, and Hoechst (Alexa 405) imaged with a Nikon C2 confocal microscope using 40x objective in (a) bright field (b) 488 channel, (c) 405 channel, (d) 594 channel with (e) merged image. Scale bar corresponded to 10 µm.
